# Groundwater sources for the Mataranka Springs (Northern Territory, Australia)

**DOI:** 10.1038/s41598-021-03701-1

**Published:** 2021-12-20

**Authors:** Sébastien Lamontagne, Axel Suckow, Christoph Gerber, Alec Deslandes, Cornelia Wilske, Steven Tickell

**Affiliations:** 1 CSIRO Land and Water, Waite Laboratories, Urrbrae, South Australia 5064 Australia; 2 Faculty of Sciences, School of Physical Sciences, The University of Adelaide, Adelaide, South Australia 5000 Australia; 3 Northern Territory Department of Environment, Parks and Water Security, Palmerston, Northern Territory 0830 Australia

**Keywords:** Environmental sciences, Hydrology

## Abstract

The Mataranka Springs Complex is the headwater of the iconic Roper River of northern Australia. Using environmental tracers measured in springs and nearby boreholes, the origin of groundwater contributing to the springs was evaluated to help assess the impact of proposed groundwater extraction in the Cambrian Limestone Aquifer (CLA) for irrigation agriculture and for hydraulic fracturing in the Beetaloo Sub-basin (an anticipated world-class unconventional gas reserve). Major ions, Sr, ^87^Sr/^86^Sr, δ^18^O-H_2_O, δ^2^H-H_2_O, ^3^H, ^14^C-DIC were consistent with regional groundwater from the Daly and Georgina basins of the CLA as the sources of water sustaining the major springs (Rainbow and Bitter) and one of the minor springs (Warloch Pond). However, ^3^H = 0.34 TU in another minor spring (Fig Tree) indicated an additional contribution from a young (probably local) source. High concentrations of radiogenic ^4^He (> 10^–7^ cm^3^ STP g^–1^) at Rainbow Spring, Bitter Spring and in nearby groundwater also indicated an input of deeper, older groundwater. The presence of older groundwater within the CLA demonstrates the need for an appropriate baseline characterisation of the vertical exchange of groundwater in Beetaloo Sub-basin ahead of unconventional gas resource development.

## Introduction

**Figure 1:**
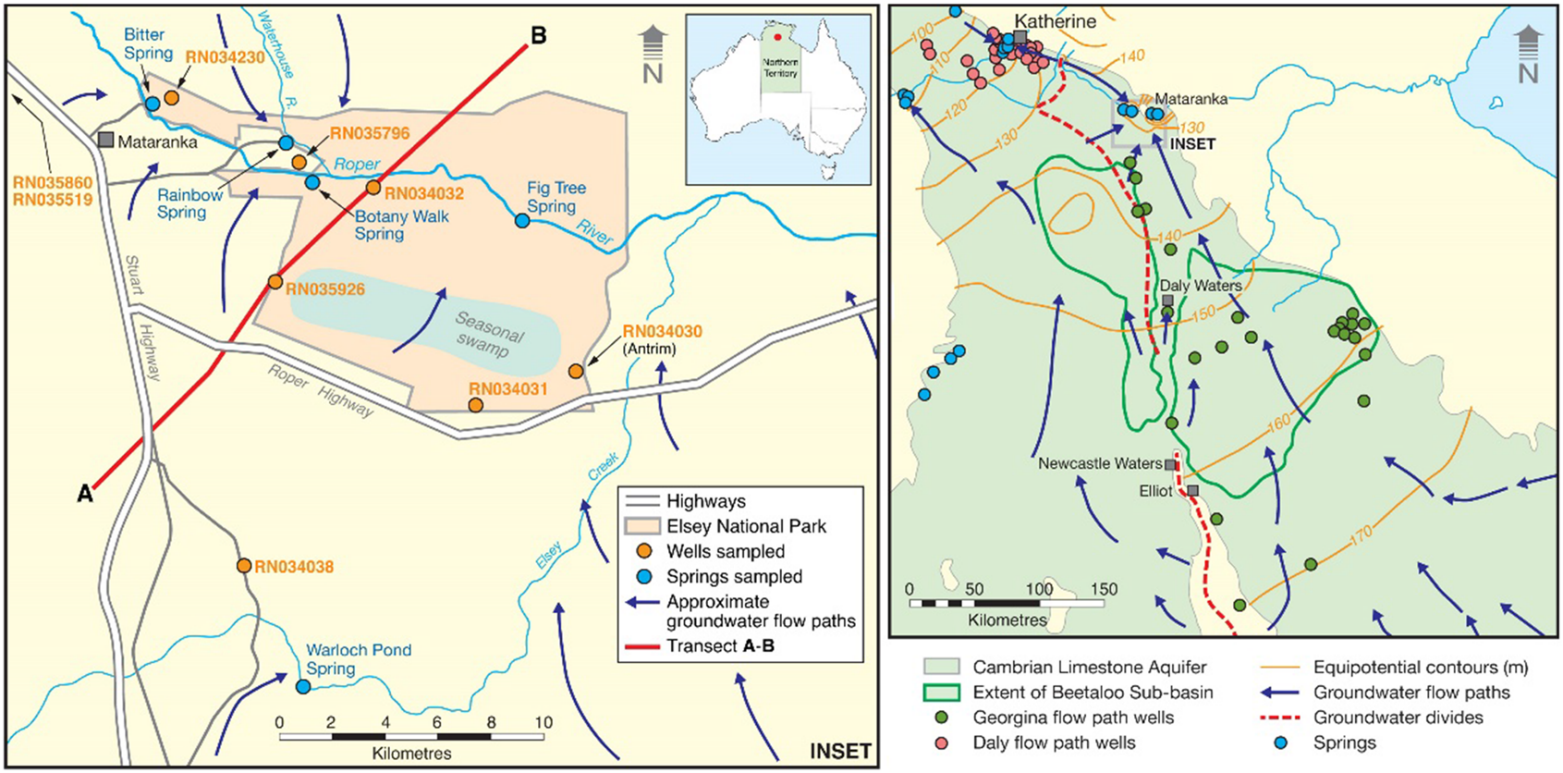
Mataranka Springs Complex, Northern Territory. The complex is a regional groundwater discharge zone for the Cambrian Limestone Aquifer, via a flow path from the north in the Daly Basin (Daly flow path), where the springs are also located, and another from the south in the Georgina Basin (Georgina flow path)^2^. Geological cross-section along A-B shown on Fig. 2. The Beetaloo Sub-basin of the Georgina Basin is to the south of the springs (see Fig. 2 for details). The springs and groundwater wells sampled in this study are all located in the vicinity of the Mataranka Springs Complex (inset). For major ions and stable isotopes of water, literature values from the Georgina and Daly basins were also used to characterise the signature for the Georgina and Daly basin groundwater flow paths (right; Table S2). Equipotential contours in m Australian Height Datum (AHD).

**Figure 2:**
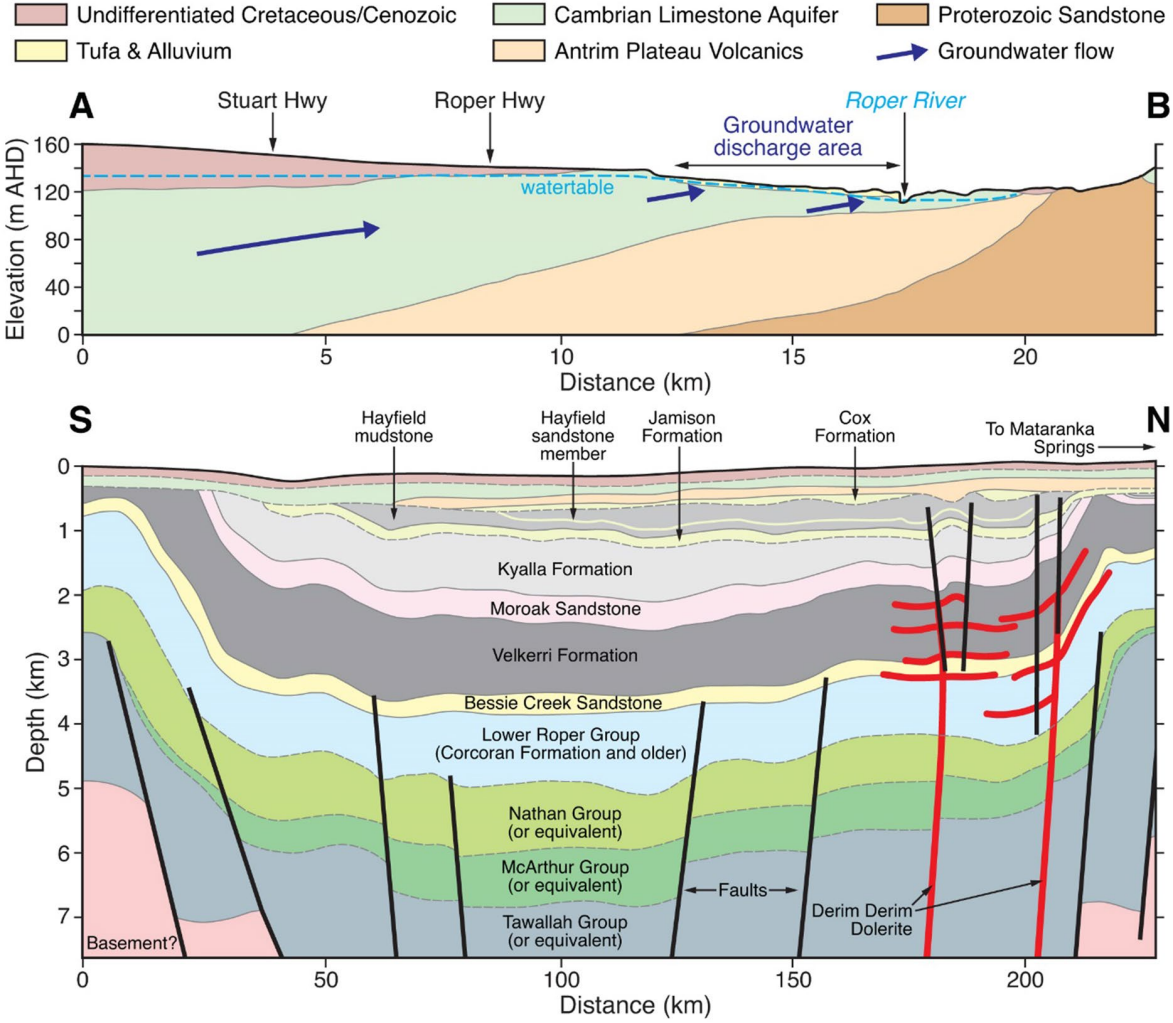
Geological cross-section of the Daly Basin near the Mataranka Springs Complex (top) (A–B transect in Fig. 1; modified from Tickell^8^) and conceptualised geological cross-section for the Beetaloo Sub-basin of the Georgina Basin (bottom; modified from Orr et al.^9^). Note the large difference in the vertical scales between the top (m Australian Height Datum) and lower cross-sections (km below ground level). The Cenozoic/Cretaceous, CLA, and Antrim Plateau Volcanics geological units (the three topmost units in the lower cross-section) are continuous from the Georgina Basin to the Daly Basin. The Proterozoic Sandstone and Siltstone of the Daly Basin (top cross-section) may be equivalent to the Bessie Creek Sandstone in the Georgina Basin (bottom cross-section) but these units are not thought to form a continuous aquifer, being isolated by a basement rise south of Mataranka.

The Cambrian Limestone Aquifer (CLA) is an extensive (474,000 km^2^) regional groundwater system and provides water resources for social, economic and cultural activities for regional areas of the Northern Territory of Australia (NT)^1–3^. Additional groundwater extraction from the CLA is being considered to help promote economic development, including for hydraulic fracturing in Beetaloo Sub-basin, anticipated to be a world-class unconventional gas reserve^4,5^. The CLA has several distinct groundwater flow systems and, for hydraulic fracturing in Beetaloo Sub-basin, groundwater would be extracted from its Georgina flow path (Fig. 1). This flow path originates from the more arid interior of the NT and flows northward to discharge into the Mataranka Springs Complex. These springs are also thought to be sustained by a shorter flow system from the north (the Daly flow path) originating at a groundwater divide between Katherine and Mataranka (Fig. 1). The CLA is exposed in the vicinity of the Mataranka Springs Complex (Fig. 2) and has numerous karst features (i.e. sinkholes) suggesting that a local recharge near the springs could also contribute to the spring flow. Whether the springs are also sustained by groundwater from geological formations below the CLA is unknown but also possible as several potential pathways for enhanced vertical hydraulic connectivity are present in the vicinity of the Mataranka Springs Complex and within the Beetaloo Sub-basin (Fig. 2). The Mataranka Springs Complex hosts a unique groundwater-dependent ecosystem, is one of the most popular tourist attractions in the NT and has a significant cultural value to the local aboriginal communities^6,7^. Thus, understanding the sources of water sustaining the springs is necessary to plan sustainable groundwater extraction from the CLA.

Here, the hydrogeological conceptual model for the Mataranka Springs Complex was tested by sampling the springs and local groundwater for major ions, Sr, ^87^Sr/^86^Sr, δ^2^H-H_2_O, δ^18^O-H_2_O, ^3^H, ^14^C-DIC and stable noble gases (^3^He, ^4^He, Ne, Ar, Kr, Xe). Sampling occurred in October 2019 (at the end of the dry season) and included the two major springs (Bitter Spring and Rainbow Spring), two minor ones (Fig Tree Spring and Warloch Pond Spring; Fig. 1) and nine groundwater samples within or close to the complex (eight from the CLA and one from the underlying Antrim Plateau Volcanics basalt; Supplementary Material Table S1). The environmental tracer database collected in this study can be found at: https://data.gov.au/data/dataset/3aa021a8-f4cf-45cb-abf1-63742efb48df. For major ions and the stable isotopes of water, trends within the complex were also compared to regional values in groundwater from the CLA (Supplementary Material Table S2). One additional minor spring (Botany Walk Spring) was too dry to sample in October 2019 but previous major ions measurements were used.

## Results

### Major ions

**Figure 3:**
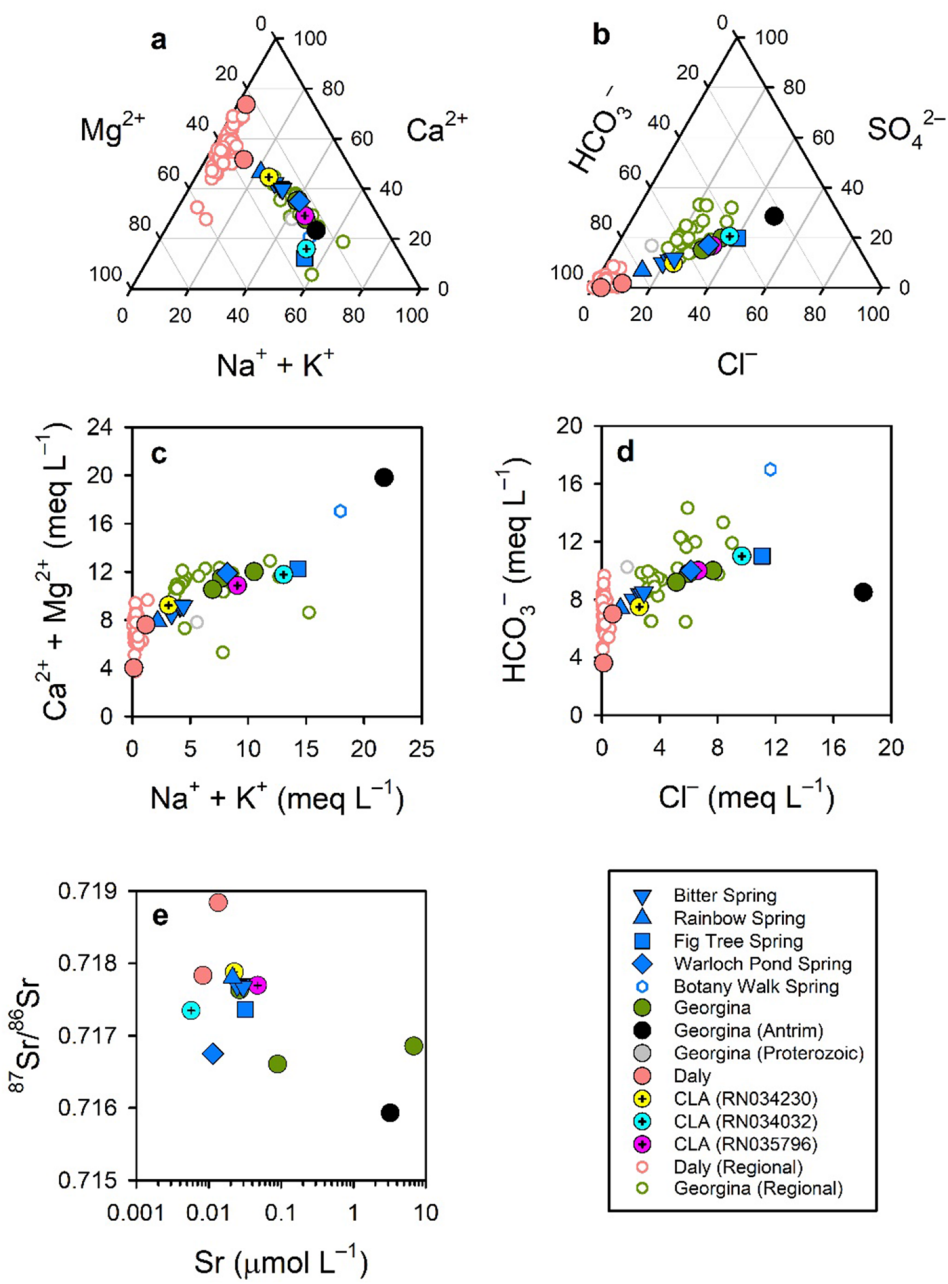
Major ion composition for springs and groundwater samples at the Mataranka Spring Complex and regionally (open symbols; Table S2) for the Georgina and Daly flow paths of the CLA. **(a)** Ternary plot for major ions; **(b)** ternary plot for major anions; **(c)** biplot for mono- relative to divalent cations; **(d)** Cl^–^ and HCO_3_^–^ biplot; **(e)** Sr and ^87^Sr/^86^Sr. Open symbols are for regional groundwater samples in the Daly and Georgina basins of the CLA and closed symbols are for samples collected in the Mataranka Springs Complex.

Water quality varied from Ca–Mg–HCO_3_ to Na–Cl dominant (Figs. 3a-d). In general, groundwater from the Daly flow path had a lower salinity and was Ca–Mg–CO_3_ dominant (that is, was > 80% Ca + Mg and HCO_3_ for cations and anions, respectively), whereas groundwater from the Georgina flow path had an overall greater salinity and a more varied major ion content (with Na + K and Cl > 20% for cations and anions, respectively). Salinity markedly increased from west to east within the complex, with the highest salinities observed in Fig Tree and Botany Walk springs and nearby groundwater. In general, the major ion composition for Rainbow Spring was most similar to the Daly flow path groundwater whilst the other springs were more similar to Georgina flow path groundwater. The highest salinity measured was in borehole RN034030, the only borehole located in the Antrim Plateau Volcanics within the complex. Borehole RN034230 is located in close proximity to Bitter Spring and had a nearly identical chemistry to the spring. Other boreholes located close to springs (RN035796 and RN034032) had chemistry similar to Georgina flow path groundwater. The boreholes on the northern (RN035860 and RN035519) and southern edge of the complex (RN035926, RN034031 and RN034038) were similar in ionic composition to Daly and Georgina Basin groundwater, respectively.

Most spring and groundwater samples in the complex were depleted in Sr (0.005–1 µmol L^–1^) relative to regional CLA groundwater elsewhere (typically > 1 µmol L^–1^; Fig. 3e). There was a distinct ^87^Sr/^86^Sr signature between Daly (> 0.7177) and Georgina flow path groundwater (< 0.7177), with Rainbow Spring and Bitter Spring intermediate between the two (~ 0.7177). Fig Tree (0.7174) and Warloch Pond springs (0.7168) had ^87^Sr/^86^Sr within the range found for Georgina flow path groundwater. The Antrim Plateau Volcanics groundwater sample had the lowest ^87^Sr/^86^Sr (0.7159). The relatively low Sr concentrations in the complex indicate the geochemical conditions favour the precipitation of Sr minerals from groundwater. The Sr isotopic content indicate Rainbow Spring and Bitter Spring would be a mixture from the Daly and Georgina groundwater flow path whilst Fig Tree Spring and Warlock Pond Spring are sustained via the Georgina flow path.

### Stable isotopes of water

**Figure 4:**
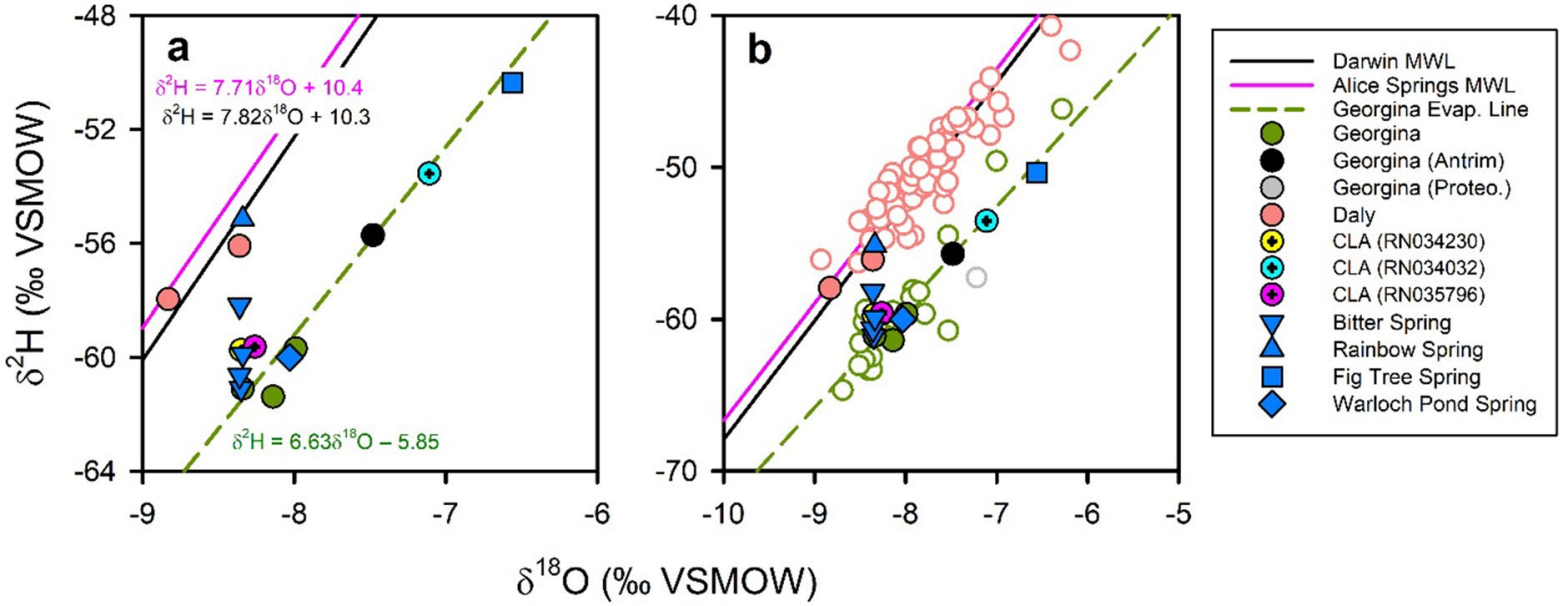
Stable isotopes of water. **(a)** Isotopic composition of springs and groundwater at the Mataranka Springs Complex, also showing the Darwin Meteoric Water Line^12^, Alice Spring Meteoric Water Line^12^ and the evaporation line for Georgina flow path groundwater. **(b)** Regional trends in isotopic composition along the Daly and Georgina flow paths (open symbols) also including groundwater and spring samples from the complex (closed symbols).

Two groups of samples can be distinguished based on δ^2^H and δ^18^O. Within the complex, Rainbow Spring and the Daly Basin flow path samples plot on the Meteoric Water Line (MWL) for either Darwin or Alice Springs (Fig. 4a), with the signature for Rainbow Spring being δ^18^O = –8.3‰ and δ^2^H = –55‰. In contrast, all groundwater samples from the Georgina flow path and the other springs fell on a distinct line consistent with evaporative enrichment of a more depleted rainfall source (δ^18^O = –14‰ and δ^2^H = –97‰). Within the complex, Bitter Spring, Warloch Pond Spring and most Georgina groundwater samples had a similar isotopic signature (approximately δ^18^O = –8.2‰; δ^2^H = –62‰). Fig Tree Spring (δ^18^O = –6.6‰; δ^2^H = –50‰) and two groundwater samples (RN034030 and RN034032) from bores in the eastern section of the study area had more evaporative enrichment. However, they were still within expectations relative to regional samples from elsewhere along the Georgina flow path (Fig. 4b). The presence of evaporative enrichment in groundwater is a common feature of arid regions and is consistent with the Georgina flow path being in a more arid section of the CLA relative to the Daly flow path^10–12^. The inferred lower isotopic signature for rainfall in the Georgina Basin is probably due to a continental effect^10^ (rainfall tends to become more isotopically depleted inland) and an amount effect^13,14^ (groundwater recharge in arid climates mostly occurs following very large rain events, which tend to be more isotopically-depleted). Whilst data are limited, the isotopic composition of groundwater samples from the Antrim Volcanics and the Proterozoic Sandstone within or south of the complex were within expectations for the Georgina flow path of the CLA.

### Tritium, ^14^C and ^4^He

**Figure 5:**
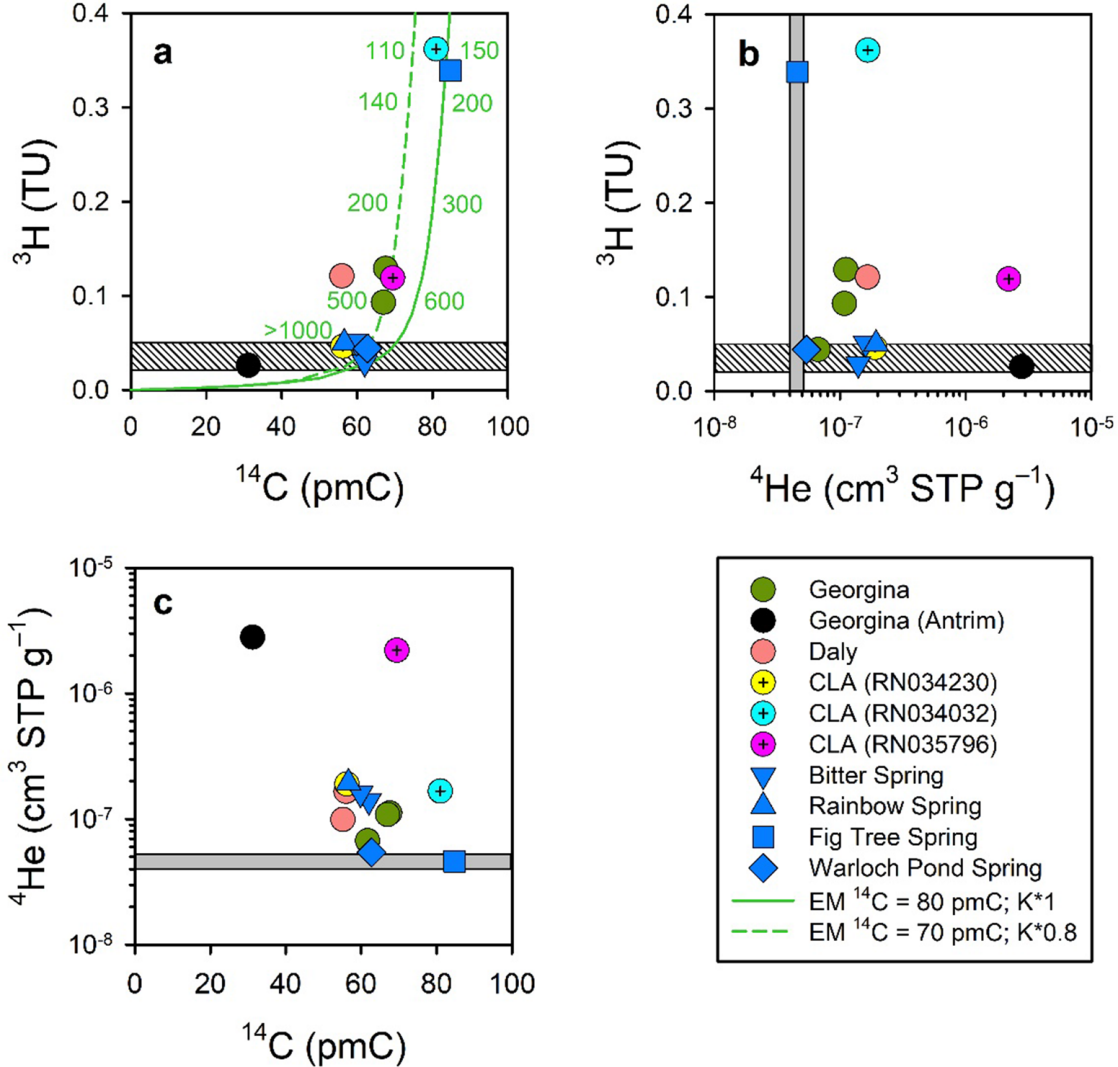
Trends in age-dating tracers in spring and groundwater. **(a)** ^14^C and ^3^H, also showing the Exponential Model (EM) for two ‘dead’ C correction factors and two formulations of the Kaitoke (K) ^3^H input function; **(b)** ^4^He and ^3^H; **(c)** ^14^C and ^4^He. The vertical grey boxes represent the range in ^4^He concentration expected for a CLA groundwater sample that would be at equilibrium with the atmosphere (i.e. that has no radiogenic ^4^He). The horizontal stripped boxes represent the analytical detection limit for ^3^H.

The examination of age-dating tracers shows a wide mixture in springs and groundwater and a contribution from a deeper, non-CLA groundwater source (or sources). Most springs had ^3^H < 0.05 TU (that is, were at the analytical detection limit), demonstrating a large component of pre-1950 water (Fig. 5a). However, Fig Tree Spring and RN034032 had ^3^H = 0.34 and 0.36 TU, respectively, showing that they contained a significant component of post-1950 water (Darwin rainfall having ~ 1.2 to 1.6 TU^15^ and the half-life of ^3^H being 12.4 years^10^). This was also consistent with a more modern ^14^C (81 pmC) in springs and groundwater where ^3^H was highest (Fig. 5a) relative to less modern values elsewhere (31–69 pmC). For ^14^C and ^3^H, the age distribution was consistent with an Exponential Model^16^, with inferred mean residence times ranging from 120 to 170 years for Fig Tree Spring to > 1000 years for the other springs (Fig. 5a).

Most spring and groundwater samples had radiogenic ^4^He (that is, had ^4^He concentrations in excess of atmospheric equilibrium, 4–5 × 10^–8^ cm^3^ Standard Temperature and Pressure (STP) g^–1^) with concentrations ranging from 4.6 × 10^–8^ to 2.6 × 10^–6^ cm^3^ STP g^–1^ (Figs. 5b,c). The highest ^4^He concentration was found in the Antrim Plateau Volcanics groundwater sample. Whilst some accumulation of radiogenic ^4^He during groundwater transport in the CLA is possible, the very high ^4^He concentrations observed in many samples combined with the large spatial variability indicated a localised input of older groundwater from an underlying confined aquifer (or aquifers). However, as the ^3^He/^4^He ratio indicated admixture of pure radiogenic helium in all samples this deeper source was not derived from primordial mantle fluids^17^.

### Other noble gases

**Figure 6:**
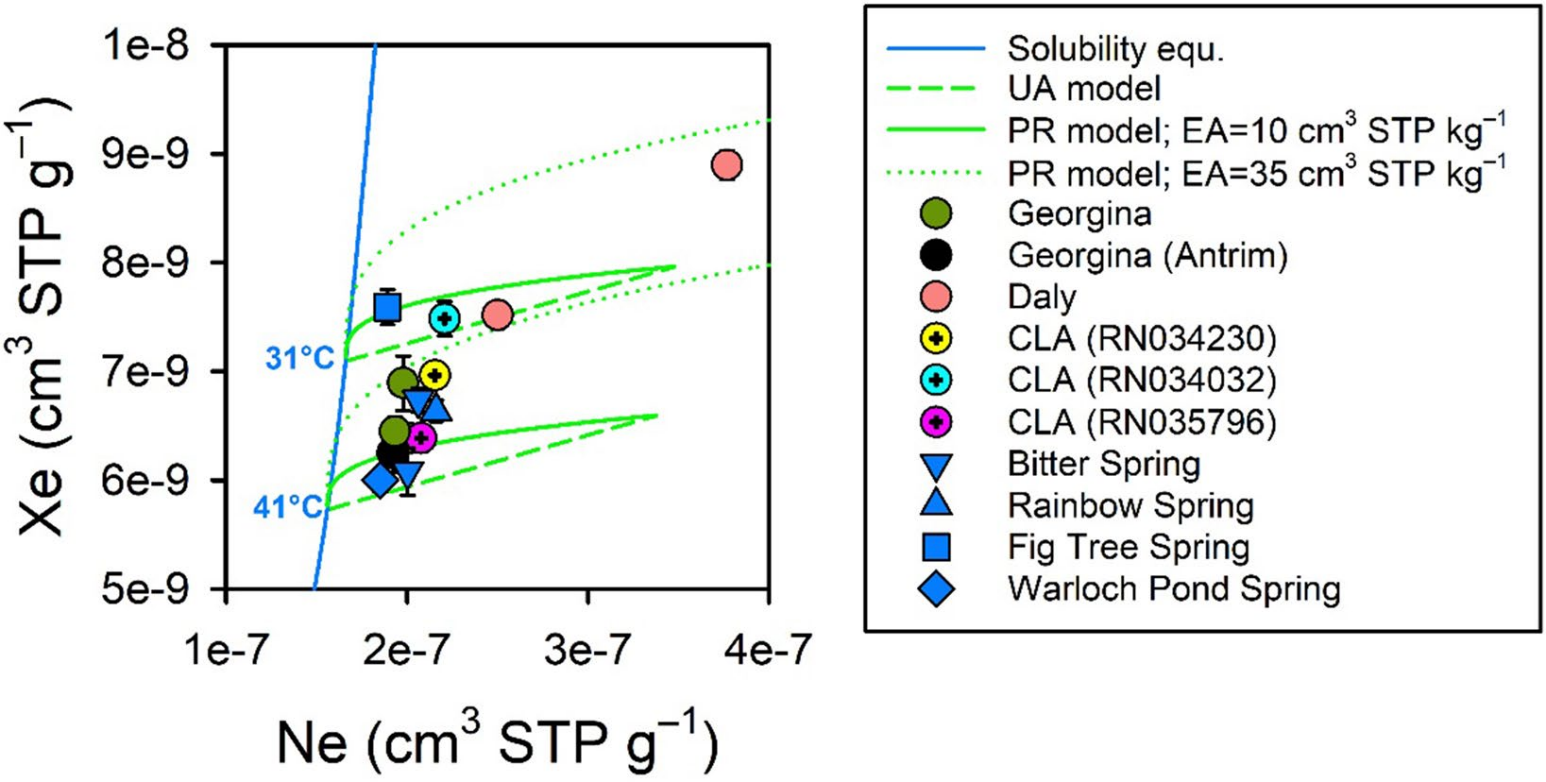
Ne and Xe trends at the Mataranka Springs Complex, showing that the relative warm temperatures of the springs are caused by the prevailing warm climate. Deviations from solubility equilibrium (blue line) are due to the presence of excess air (EA) in groundwater. The green lines represent the possible range in noble gas concentrations at a given recharge temperature using either the unfractionated excess air model (UA) or the Partial Re-equilibration (PR) excess air model (for two initial EA contents). Mean shown with ± one standard deviation.

The springs in the complex are notable for being ‘warm’ (25–33 °C). The trends in noble gases (here shown through Ne and Xe) indicate the warm temperatures are due to a relatively high ambient soil temperature at the time of recharge rather than from geothermal heating (Fig. 6). All samples had a relatively large amount of excess air (that is, Ne and Xe concentrations were greater than expectations from solubility equilibrium). Using a combination of the unfractionated excess air model (UA) and the partial re-equilibration model (PR) for a moderate (10 cm^3^ STP kg^–1^) and a large (35 cm^3^ STP kg^–1^) amount of excess air^18,19^, the recharge temperature would range from 31 to 41 °C. The Daly flow path samples had a greater amount of excess air and a lower inferred recharge temperature than the Georgina flow path groundwater samples. Fig Tree Spring had the lowest amount of excess air and the lowest inferred recharge temperature. This suggests extensive re-equilibration with the atmosphere on a seasonal basis caused by groundwater flow through large, partially saturated cavities in the tufa. As sampling occurred at the end of the (relatively cooler) dry season, the inferred temperature at the time of recharge would appear colder if groundwater discharging at Fig Tree Spring had recently undergone partial re-equilibration. This could be further evaluated by measuring the changes in the noble gas content of the springs on a seasonal basis.

## Discussion

### Sources of water for the Mataranka Springs complex

**Figure 7:**
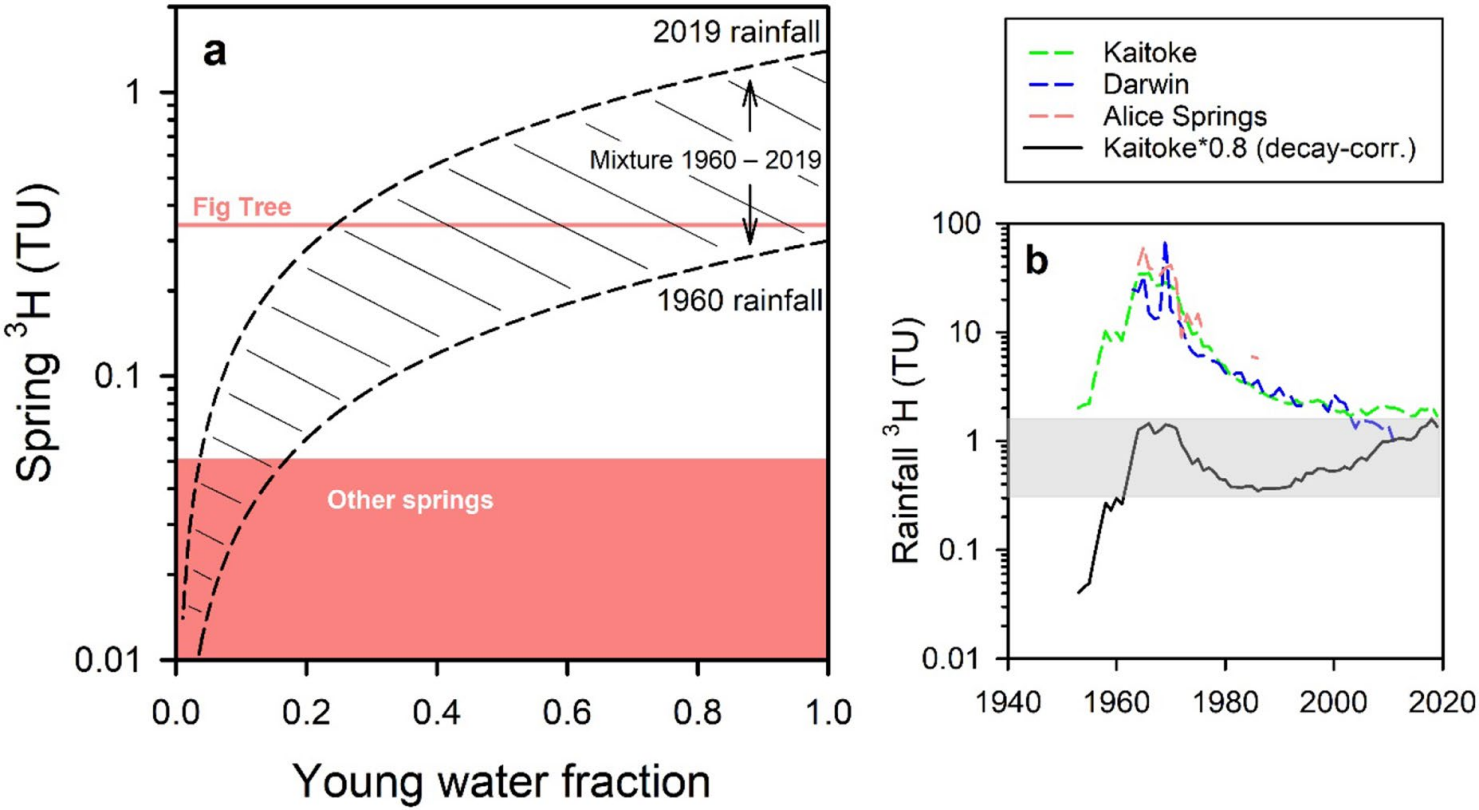
**(a)** Estimation of the ‘young’ groundwater component in the springs based on **(b)** the decay-corrected ^3^H content for rainfall between 1960 and 2019 (shaded area). As ^3^H is near analytical detection limit in springs other than Fig Tree, the young age fraction is an upper estimate for them. The envelope of potential admixtures of young and old water also considers most potential age-distributions within the young fraction.

The original conceptual model from Karp^20,21^ was for the Mataranka Springs Complex to be fed via the Daly and the Georgina regional flow paths of the CLA. However, a component of local recharge feeding the springs was also anticipated owing the less permeable Cretaceous sediment cover overlying much of the CLA elsewhere being absent in the area surrounding Elsey National Park, which should facilitate local recharge processes. The findings of this study are largely consistent with Karp. Based on salinity, ionic composition, δ^2^H and δ^18^O, Rainbow Spring is primarily fed by the Daly flow path of the CLA whilst Bitter Spring, Warloch Pond Spring and Fig Tree Spring are primarily fed by the Georgina flow path of the CLA. The regional scale of the flow systems is also reflected in the mixture of groundwater ages in the springs^16^. Tritium > 0.1 TU in Fig Tree Spring and in several groundwater samples also demonstrated a contribution from recent (and probably local) recharge to the Georgina flow path of the CLA, especially at the eastern end of the complex. A simple end-member mixing analysis indicated the young fraction component was at least 20% of Fig Tree Spring flow but negligible to less than 18% at the other springs (Fig. 7). Thus, most of the groundwater sustaining the major springs (Rainbow and Bitter) is regional in nature, that is originates from recharge a significant distance away from the springs.

A contribution from a deeper source (or sources) inferred from radiogenic ^4^He had not been demonstrated previously for the Mataranka Springs Complex. As radiogenic ^4^He was more elevated in Rainbow and Bitter springs, the deeper source(s) may also contribute to the baseflow of the Roper River. The timescale of regional groundwater transport within the CLA is too short (< 10,000 years) and the U and Th content of the limestone too low^22^ to account for the elevated radiogenic ^4^He content. However, the timescale of groundwater transport in underlying formations is unknown but anticipated to be much longer than in the CLA, which would foster the accumulation of radiogenic ^4^He. This is consistent with the ^4^He > 10^–6^ cm^3^ STP g^–1^ measured in Antrim Plateau Volcanics groundwater. There may also be a contribution from other geological formations underlying the CLA owing the presence of faults in the region^9^, including faults that have been reactivated in the recent geological history^23^. The concentrations of radiogenic ^4^He found at the Mataranka Springs Complex are within the range (10^–8^ to 10^–4^ cm^3^ STP g^–1^) found in large confined sedimentary aquifers in Australia^24–26^. It is not possible to quantify the contribution from the deeper groundwater source(s) at present because there is very limited information about tracer signatures for groundwater in any of the geological formations underlying the CLA.

The evidence for partial re-equilibration of the gas content with the atmosphere for some spring and groundwater samples indicates the radiogenic ^4^He content provided here is conservative (that is, some of the initial radiogenic ^4^He may have been lost during groundwater transit through the complex). The noble gas-inferred recharge temperatures are broadly consistent with the measured spring temperatures, which indicates the deeper groundwater source is not geothermally-heated or that its contribution is small relative to the one from the CLA. A more quantitative interpretation of the source of radiogenic ^4^He will require the deeper formations in the complex and in Beetaloo Sub-basin to be instrumented and for near-surface measurements to be carefully corrected for the presence of excess air and for partial re-equilibration effects with the atmosphere.

### Salt balance

Deeper groundwater discharge and extensive groundwater transpiration may both contribute to the increase in groundwater salinity observed in parts of the Mataranka Springs Complex. Whilst springs are the more obvious groundwater discharge feature in the landscape, there is significant groundwater evapotranspiration throughout the complex, consistent with a net annual evaporative flux estimated by remote sensing (~ 250 mm year^–1^)^27^. This net evaporative flux would tend to increase the salinity of shallow groundwater, as is commonly observed in other regional groundwater discharge zones in arid environments^28,29^. Alternatively, the increase in groundwater salinity in the complex could be caused by saline groundwater discharge to the CLA from a deeper groundwater source (or sources). The two processes are not mutually exclusive and deeper groundwater discharge may also be partially transpired when occurring in areas with shallow water tables within the Complex. Thus, better constraining the contribution of the various groundwater sources to the springs will require both defining the geochemical signature of the deeper groundwater source(s) and an independent assessment of groundwater transpiration by phreatophytes within the Complex.

### Implications for water resources development

The potential impact of groundwater extraction in the CLA on the Mataranka Springs Complex will be commensurate with the extraction rate, the distance of the extraction from the complex, and the nature of the interaction between groundwater and the various groundwater-dependent assets within the complex^30^. Recent evaluations of proposed groundwater extraction scenarios for the Georgina Basin of the CLA suggest a potential 20% decline of the Roper River baseflow over 300 years^31^. However, as groundwater—surface water interactions in the Mataranka Springs Complex are not represented in current hydrogeological models of the CLA, the potential impacts of planned extraction on springs and other groundwater-dependent assets are unclear.

The potential for hydraulic fracturing to increase the vertical connectivity between water resources and deeper geological formations is a key concern of the public relative to unconventional gas resources development in Beetaloo Sub-basin^32^ and elsewhere^33,34^. Demonstrating the presence of a deeper groundwater source at the springs (and for the CLA in general^24^) is a key requirement for pre-development baseline assessments for unconventional gas resources^35–37^. Whilst it was established here that at least one deeper groundwater source is present pre-development at the springs, the exact origin of the deeper source (or sources), the mode of connectivity with the CLA (faults, contiguous formations, etc.) and the proportion of the spring flow attributable to deeper groundwater could not be evaluated because of the lack of infrastructure (that is, monitoring boreholes) in the geological formations underlying the CLA^24^. Due to a significant interburden thickness, hydraulic fracturing in Beetaloo Sub-basin is unlikely to materially increase the vertical hydraulic connectivity between rocks hosting unconventional gas plays and the CLA^38^. Nevertheless, additional baseline geochemical mapping and a field evaluation of the existing pathways for vertical connectivity are required to guide the development of unconventional gas resources in the Beetaloo Sub-basin^37,39,40^.

## Methods

### Study area

Climate in the study area ranges from tropical savanna near Mataranka and Katherine to hot semi-arid further south, with a distinct north–south monsoon-influenced rainfall gradient. At Katherine (Bureau of Meteorology Station 014903), annual precipitation varies from 678 to 1575 mm, annual potential evaporation is 2227 mm, and the average daily maximum temperature is 34 °C. The hydrogeology of the CLA is complex owing to the north–south rainfall gradient, distinct geological basins (Wiso, Daly and Georgina) with their own groundwater flow systems, extensive karst features, partial confinement, and the potential for vertical hydraulic connectivity with underlying geological units. At the Mataranka Springs Complex, the CLA is unconfined, relatively thin, but overlain by a layer of tufa (redeposited limestone) with extensive karst features (Fig. 2). Two local confined aquifers, the Antrim Plateau Volcanics basalt and the Proterozoic Sandstone and Siltstone, underlie the CLA near Mataranka and outcrop to the north of the study area. The extent of tufa deposition (> 10 m) at the Mataranka Springs Complex indicates the area has been a regional groundwater discharge zone for millennia. The Beetaloo Sub-basin underlies part of the Georgina Basin and is approximately 150 km south from Mataranka (Fig. 1).

### Environmental tracer sampling

Spring and groundwater samples were collected from the Mataranka Springs Complex between 16 and 20 October 2019. The locations of the springs and boreholes and the borehole construction details can be found in Table S1. Note that borehole RN034030 located in the Antrim Plateau Volcanics basalt was the only borehole available in the area that had a screen located in a geological formation other than the CLA. The two largest springs (Rainbow Spring and Bitter Springs) flow at 0.3–0.8 m^3^ s^–1^ and generate short streams popular as tourist attractions before they cascade into the Roper River^20^. Rainbow Spring has one cluster of vents over approximately a 10 m^2^ area generating the streamflow, whereas Bitter Springs appears to have many vents (> 10) spread over a larger, densely vegetated area (~ 1000 m^2^) joining together to form a stream. Warloch Pond Spring is one of probably many vents discharging into Elsey Creek, whereas Fig Tree Spring discharges horizontally from a cavity in a tufa cliff along the riparian corridor of the Roper River. These smaller springs have not been gauged previously but are estimated to have a discharge < 0.1 m^3^ s^–1^.

To avoid degassing losses, the springs were sampled by either installing a small submersible pump in a vent or, at Fig Tree Spring, by inserting a tube in the cavity. Samples were collected from one of the Rainbow Spring vents, two of the Bitter Springs vents and from the Fig Tree and Warloch Pond Spring vents (with, for selected analytes, two additional surface water samples collected from the Bitter Spring stream downstream from the vents). For groundwater samples, a submersible pump was lowered to the screen interval and the borehole casing was dewatered for at least three bore volumes before sample collection was initiated. A YSI™ multi-parameter probe (https://www.ysi.com) was used to measure pH, specific electrical conductance (EC), temperature and dissolved oxygen concentrations. For major and minor elements, samples were filtered through a 0.45 µm nitrocellulose membrane filter and (for cations) acidified with (1% v/v HNO_3_). Alkalinity was measured in the field using a HACH™ titration kit (https://www.hach.com). Delta-^18^O and δ^2^H samples were stored in 28 mL gas-tight glass bottles (McCartney). Tritium, δ^13^C-Dissolved Inorganic Carbon (DIC) and ^14^C-DIC samples were stored in 1 L HDPE bottles, unfiltered with no headspace and with no preservative. Sr (and strontium isotopes) samples were collected unfiltered in 125 mL plastic bottles. Spring and groundwater samples for noble gases were collected using the copper tube method^41^.

Major and minor cations were analysed by a SPECTRO CIROS Radial Inductively Coupled Plasma Optical Emission Spectrometer and anions using a Dionex ICS-2500 Ion Chromatrograph at CSIRO Land & Water Analytical Services, Adelaide, South Australia. The charge balance error on the major ion measurements was ± 5%. Strontium and ^87^Sr/^86^Sr were measured by Inductive-Coupled Mass Spectrometry at the CSIRO Land & Water Analytical Services. Stable isotopes of water were measured by Isotope Ratio Mass Spectrometry at GNS, New Zealand, with a precision of 0.1‰ and 1.0‰, for δ^18^O and δ^2^H, respectively. Noble gases were measured at the CSIRO Land & Water Noble Gas Laboratory using offline separation of gases from water by cryogenic techniques, separation of reactive gases from noble gases using catalyst and getter systems and separation of the noble gas fractions using cryogenic techniques down to 13 K. Total gas content was evaluated using a spinning rotor gauge, the isotopic composition using quadrupole mass spectrometers and a high-resolution Helix MC noble gas mass-spectrometer^42^. Tritium was measured by electrolytic enrichment and liquid scintillation counting with a detection limit of 0.025 TU^43^. Radiocarbon and δ^13^C-DIC were measured by accelerator mass spectrometry and Isotope Ratio Mass Spectrometry at the Rafter Radiocarbon Laboratory with an accuracy of 0.2‰ for δ^13^C and a detection limit of 0.5% Modern Carbon (pmC) for ^14^C.

### Lumped-parameter models

The age-distribution of spring and groundwater was evaluated using lumped-parameter models^16,44^. For simplicity, only the results of the Exponential Model (EM) are reported here for two different initial ^14^C values corrected for ‘dead’ C inputs (^14^C = 70 pmC and 80 pmC) due to the dilution of rainfall ^14^C by rock-water interactions in the aquifer. The input function for ^3^H from Kaitoke was multiplied by a factor of 0.8, which approximates the (incomplete) Darwin rainfall ^3^H record.

### Young groundwater fraction

The ‘young’ (post-1950) groundwater component at Mataranka was estimated as ^3^H_sp_ = *p*_y_·^3^H_dc_, where ^3^H_sp_ is the spring ^3^H, *p*_y_ the proportion of post-1950 water in spring water and ^3^H_dc_ is the decay-corrected value for rainfall for a particular year (with the pre-1950 component assumed to be ^3^H-free). For simplicity, an envelope of potential ^3^H_sp_ was estimated using a relatively low ^3^H_dc_ (1960; 0.3 TU) and high ^3^H_dc_ (2019; 1.4 TU). This envelope also describes most age-distributions within the post-1950 component.

## Supplementary Information

Supplementary Table S1.

Supplementary Table S2.

## Data Availability

Spring and borehole location can be found in Supplementary Material Table S1. The environmental tracer dataset collected in this study can be found at https://data.gov.au/data/dataset/3aa021a8-f4cf-45cb-abf1-63742efb48df. Supplementary Material Table S2 provides the additional literature data used to characterise the major ions and stable isotopic signature of Daly and Georgina basins groundwater (Botany Walk Spring: Mean of three samples from Karp^20^). The summary for major elements, ^87^Sr/^86^Sr, U and Th content from rocks in the Georgina flow path of the CLA can be found at https://data.gov.au/data/dataset/49e25e37-ab1c-481a-9ad9-84ec201a9dc7. The Darwin and Alice Springs rainfall ^3^H time series is available upon request from gnip@ansto.gov.au and the Kaitoke ^3^H time series was obtained by personal communication from Uwe Morgenstern, GNS New Zealand.
