# Supplementary material for: Groundwater sources for the Mataranka Springs (Northern Territory, Australia)
Source: Sci Rep. 2021 Dec 20;11:24288. doi: 10.1038/s41598-021-03701-1 (PMC8688415; doi:10.1038/s41598-021-03701-1)
Supplement: Supplementary file 1 — Supplementary Table S1. [file 41598_2021_3701_MOESM1_ESM.docx]

**Supplementary Material**

Lamontagne et al. Groundwater sources for the Mataranka Springs (Northern Territory, Australia)

**Table S1.** Spring and bore location with bore construction details. Additional bore details can be found by consulting drilling reports at NR Maps (NRmaps.nt.gov.au). Standing water levels measured in October 2019 (end of dry season).

| Bore/Spring | Longitude | Latitude | Screen Interval  (m below ground surface) | Geological formation where screen is located | Inferred regional flow path based on hydraulic gradients relative to Roper River | Ground elevation (m Australian Height Datum) | Standing water level (m below ground surface) | Other |
| --- | --- | --- | --- | --- | --- | --- | --- | --- |
|  |  | **Springs** | | | |  |  |  |
| Bitter Spring Vent 1 | 133.089430 | -14.911900 |  |  |  |  |  |  |
| Bitter Spring Vent 2 | 133.089940 | -14.912110 |  |  |  |  |  |  |
| Bitter Stream 1 | 133.089968 | -14.913484 |  |  |  |  |  |  |
| Bitter Stream 2 | 133.089815 | -14.912528 |  |  |  |  |  |  |
| Rainbow Spring | 133.134723 | -14.922594 |  |  |  |  |  |  |
| Fig Tree Spring | 133.215352 | -14.951150 |  |  |  |  |  |  |
| Warloch Pond Spring | 133.137020 | -15.111020 |  |  |  |  |  |  |
|  |  | **Bores** | | | |  |  |  |
| RN034030 | 133.197490 | -15.016040 | 27 – 29 | Antrim Plateau Volcanics (basalt) | Contiguous to the Georgina Basin flow path of the CLA | 125.65 | 2.15 | Near interface with Tindall Limestone |
| RN034031 | 133.233170 | -15.002260 | 35 – 41 | Tindall Limestone | Georgina | 136.61 | 4.80 |  |
| RN034032 | 133.092930 | -14.903610 | 9.5 – 16 | Tindall Limestone/tufa | Undefined | 123.45 | 7.85 | Near interface with basalt |
| RN034038 | 133.124500 | -15.083700 | 11 – 14 | Tindall Limestone | Georgina | 134.53 | 1.65 |  |
| RN034230 | 133.092930 | -14.903610 | 12 – 18 | Tindall Limestone | Undefined | 130.93 | 3.42 |  |
| RN035519 | 133.002370 | -14.868380 | 33 – 35 | Tindall Limestone | Daly | 139.34 | 8.52 |  |
| RN035796 | 133.138180 | -14.931910 | 20 – 26 | Tindall Limestone | Undefined | 121.58 | 4.70 | Near interface with basalt |
| RN035860 | 132.823520 | -14.724220 | 46 – 53 | Tindall Limestone | Daly | 156.60 | 22.28 |  |
| RN035926 | 133.129940 | -14.971540 | 16 – 22 | Tindall Limestone | Georgina | 132.97 | 2.10 |  |
